# A new scoring system for predicting survival in patients with non-small cell lung cancer

**DOI:** 10.1002/cam4.479

**Published:** 2015-06-23

**Authors:** Steven E Schild, Angelina D Tan, Jason A Wampfler, Helen J Ross, Ping Yang, Jeff A Sloan

**Affiliations:** 1 Department of Radiation Oncology, Mayo Clinic5881 E. Mayo Blvd, Phoenix, Arizona, 85054; 2 Division of Biomedical Statistics and Informatics, Mayo Clinic200 First St S.W., Rochester, Minnesota, 55905; 3 Division of Medical Oncology, Mayo Clinic13400 E. Shea Blvd., Scottsdale, Arizona, 85259; 4 Epidemiology-Cancer Research200 First St S.W., Rochester, Minnesota, 55905; 5 Cancer Center Statistics, Mayo Clinic200 First St S.W., Rochester, Minnesota, 55905

**Keywords:** Age, metastases, nodal spread, non-small cell lung cancer, performance status, prognosis, quality of life, scoring system, smoking, tumor size

## Abstract

This analysis was performed to create a scoring system to estimate the survival of patients with non-small cell lung cancer (NSCLC). Data from 1274 NSCLC patients were analyzed to create and validate a scoring system. Univariate (UV) and multivariate (MV) Cox models were used to evaluate the prognostic importance of each baseline factor. Prognostic factors that were significant on both UV and MV analyses were used to develop the score. These included quality of life, age, performance status, primary tumor diameter, nodal status, distant metastases, and smoking cessation. The score for each factor was determined by dividing the 5-year survival rate (%) by 10 and summing these scores to form a total score. MV models and the score were validated using bootstrapping with 1000 iterations from the original samples. The score for each prognostic factor ranged from 1 to 7 points with higher scores reflective of better survival. Total scores (sum of the scores from each independent prognostic factor) of 32–37 correlated with a 5-year survival of 8.3% (95% CI = 0–17.1%), 38–43 correlated with a 5-year survival of 20% (95% CI = 13–27%), 44–47 correlated with a 5-year survival of 48.3% (95% CI = 41.5–55.2%), 48–49 correlated to a 5-year survival of 72.1% (95% CI = 65.6–78.6%), and 50–52 correlated to a 5-year survival of 84.7% (95% CI = 79.6–89.8%). The bootstrap method confirmed the reliability of the score. Prognostic factors significantly associated with survival on both UV and MV analyses were used to construct a valid scoring system that can be used to predict survival of NSCLC patients. Optimally, this score could be used when counseling patients, and designing future trials.

## Introduction

In 2013, lung cancer caused an estimated 159,480 deaths in the US 1. Approximately 85% of lung cancer patients were diagnosed with non-small cell lung cancer (NSCLC) with the majority of patients presenting with advanced disease 2. Despite gradual improvements in prognosis over time, the majority of the estimated 228,190 Americans diagnosed in 2013 with lung cancer will succumb to it. More research in the prevention, screening, and treatment of lung cancer is required to alter this dismal situation. When writing trials for lung cancer patients, it is important to have a clear understanding of the effects of pretreatment prognostic factors on outcome. This is critical to proper trial design where one optimally stratifies patients for these factors evenly between the treatment arms. This is done to prevent the introduction of uncontrolled biases that can confound the results leading to incorrect conclusions. A valid scoring system could be used to potentially improve the quality of trials performed by allowing better balance of prognostic factors between the treatment arms and the selection of high-risk patients for specific trials. Additionally, the clear understanding of prognosis can help physicians counsel patients about outcome and choose appropriate treatment for individual patients.

In this study, we evaluated the outcome of a large patient cohort to identify their pretreatment prognostic factors and created a scoring system that can stratify patients into groups with distinctly different outcomes. We also carried out validation testing of this scoring system.

## Materials and Methods

A total of 1274 patients with NSCLC from a retrospective analysis selected from more than 10,000 patients enrolled to the Mayo Clinic Epidemiology and Genetics of Lung Cancer Research Program were used to generate this scoring system. These patients were registered between 1 March 1997 and 29 april 2008 and were selected because they had complete data available regarding the prognostic factors used for this analysis. Details of the research program and the approach used for identifying and observing patients have been previously presented 3,4. In this study, we aimed to produce a valid scoring system that could be used to segregate NSCLC patients into groups with differing survival. Baseline factors examined included overall quality of life (QOL), age, treatment, sex, tumor diameter (cm), regional nodal involvement, distant metastasis, Eastern Cooperative Oncology Group (ECOG) performance score, presence of other malignancy, smoking category and status at diagnosis, years since quitting smoking, and pack-years of smoking. These factors were identified as potential prognostic factors associated with survival in the previous study 2. Weight loss of ≥5% in past 6 months was also included as this is an established prognostic factor in NSCLC 5. Patients with distant metastases included 16 patients with metastases within the other lung (M1a), seven patients with pleural nodules (M1a), four patients with pleural effusion (M1a), 1 patient with pericardial effusion (M1a), and 53 patients with distant metastases in extra-thoracic organs (M1b). Stage was specifically not used as it is changed every few years and would negate the value of this score when the staging system is redefined.

QOL was assessed with a single-item from the Lung Cancer Symptom Scale. The overall QOL item was used by Sloan et al. 4 and in this study. Overall QOL was considered as a single continuous variable, taking integer values from 0 to 100 (ranging from “as bad as it can be” to “as good as it can be”). The patients judged their own QOL and filled out this single question on a sliding scale. A score of 50 or lower was indicative of a deficit in QOL and related to patient survival.

The Cox proportional hazards model was used to assess the prognostic significance of baseline factors in UV and MV analyses 6. Those independent prognostic factors significant in both analyses were used to develop the scoring system. The 5-year overall survival (OS) rate (as the percentage) was first calculated for each level of the significant prognostic factors. The 5-year OS rate for each level was divided by 10 to obtain the corresponding score (as whole digit). For example, if patients with ECOG performance status of 0–1 had a 5-year OS rate of 62%, the corresponding score for performance status was calculated by dividing 62 by 10 resulting in a score of 6. In contrast, if patients with performance status of 2–4 had a 5-year OS rate of 24%, the corresponding score is 24/10 or 2. The sum of scores from all significant independent prognostic factors was calculated to form a total score for each patient. The median survival and 5-year OS rates for patients grouped within various ranges of total scores were calculated using Kaplan–Meier survival estimates. Categorization of the score was delineated first by clinician expert opinion and then by multiple statistically defined empirical cut points.

Bootstrapping was employed to assess the relative robustness of the model and provide preliminary evidence of validity 6. Multivariate Cox proportional hazards models were bootstrapped, wherein we took a random sample, with replacement of the same size as the original sample to obtain a MV model using stepwise selection 7. We created 1000 bootstrap samples, and obtained 1000 estimates of the MV model. We then summarized the percentage of time each variable was selected in the bootstrapped model. A similar approach was also used to validate the score for each level of prognostic factors, where Kaplan–Meier survival estimates were used to calculate the 5-year survival rate; and the basic statistics from 1000 bootstrap samples were summarized. Survival rates observed were accurate to within 2% with 95% confidence.

## Results

The most common patient group represented was white married men who were former smokers with good performance status, and early disease stage that was resected 2. Patient demographics are presented in Table1.

**Table 1:** Overall patient demographics

	Total (*N *=* *1274)
Age group	
<60	300 (23.5%)
60–69.999	427 (33.5%)
70–79.999	440 (34.5%)
≥80	107 (8.4%)
Tumor size (cm)	
*N*	1274
Mean (SD)	3.1 (2.1)
Median	2.5
Q1, Q3	1.7, 4.0
Range	0.0–19.0
Tumor size (as categorical data)	
≤2 cm	479 (37.6%)
>2 cm	795 (62.4%)
Regional nodal involvement	
No nodal metastases	927 (72.8%)
In ipsilateral peribronchial and/or ipsilateral hilar nodes	123 (9.7%)
In ipsilateral mediastinal and/or subcarinal nodes	196 (15.4%)
In contralateral mediastinal nodes	28 (2.2%)
Distant metastasis	
Absence	1193 (93.6%)
Presence	81 (6.4%)
Smoker category	
Never	229 (18.0%)
Former	695 (54.6%)
Recent quitter/abstinent	205 (16.1%)
Current/persistent	145 (11.4%)
Cell type	
Non-SCLC	1274 (100.0%)
Treatment	
Missing	36
Surgery	1108 (89.5%)
Rad or Chemo only	45 (3.6%)
Rad + Chemo	76 (6.1%)
Other	9 (0.7%)
Gender	
Female	604 (47.4%)
Male	670 (52.6%)
Race	
Caucasian	1188 (93.2%)
Hispanic	8 (0.6%)
American Indian/Alaska Native	68 (5.3%)
Black	7 (0.5%)
Asian/Pacific Islander	3 (0.2%)
ECOG performance status	
Missing	24
0 = fully active	526 (42.1%)
1 = light work	568 (45.4%)
2 = unable to work	117 (9.4%)
3 = limited self care	33 (2.6%)
4 = disabled	6 (0.5%)
Smoking cessation	
Quit	1217 (95.5%)
Kept smoking	57 (4.5%)
Pack-years smoked	
Missing	5
0–20	426 (33.6%)
20–40	280 (22.1%)
>40	563 (44.4%)
Any other cancer	
Missing	117
Yes	174 (15.0%)
No	983 (85.0%)
Any lung disease	
No	987 (77.5%)
Yes	287 (22.5%)
Any other disease	
No	930 (73.0%)
Yes	344 (27.0%)
Weight loss of 5% in past 6 months	
Missing	41
No	1070 (86.8%)
Yes	163 (13.2%)

ECOG, Eastern Cooperative Oncology Group.

In the UV analysis, age, tumor diameter, regional nodal involvement, distant metastasis, overall QOL, treatment, sex, ECOG performance score, smoking cessation, and pack-years smoked were significant prognostic factors of survival (Table2). All factors significant on UV analysis were included in MV analysis, except the treatment and pack-years of smoking. Treatment was not included as the goal was to develop a pretreatment score. The number of pack-years was excluded because it is a collinear confounding factor with smoking cessation.

**Table 2:** Univariate Cox regression model survival analysis using first QOL assessment—all patients

Variable	*N*	Events	Cox univariate hazard ratio (95% CI)	Cox univariate Wald *P*-value	Cox univariate score *P*-value
Age group					<0.0001
<60	300	83 (28%)	0.46 (0.33, 0.65)	<0.0001	
60–69.999	427	123 (29%)	0.45 (0.33, 0.62)	<0.0001	
70–79.999	440	179 (41%)	0.747 (0.55, 1.01)	0.0604	
≥80^1^	107	54 (50%)	–		
Tumor size (cm)					<0.0001
≤2 cm	479	120 (25%)	0.54 (0.44, 0.66)	<0.0001	
>2 cm^1^	795	319 (40%)	–		
Regional nodal involvement					<0.0001
No nodal metastases	927	257 (28%)	0.20 (0.13, 0.33)	<0.0001	
In ipsilateral peribronchial and/or ipsilateral hilar nodes	123	52 (42%)	0.36 (0.21, 0.62)	0.0002	
In ipsilateral mediastinal and/or subcarinal nodes	196	112 (57%)	0.59 (0.36, 0.97)	0.0381	
Metastasis in contralateral mediastinal nodes^1^	28	18 (64%)	–		
Distant metastasis					<0.0001
Absence	1193	375 (31%)	0.21 (0.16, 0.27)	<0.0001	
Presence^1^	81	64 (79%)	–		
QOL					<0.0001
Non deficit (QOL > 50)^1^	1045	315 (30%)	–		
Deficit (QOL ≤ 50)	229	124 (54%)	2.94 (2.38, 3.63)	<0.0001	
Smoker category					0.1698
Never	229	69 (30%)	0.69 (0.48, 0.98)	0.0378	
Former	695	242 (35%)	0.87 (0.65, 1.17)	0.3705	
Recent quitter/abstinent	205	74 (36%)	0.90 (0.63, 1.28)	0.5577	
Current/persistent^1^	145	54 (37%)	–		
Treatment					<0.0001
Surgery	1108	337 (30%)	0.13 (0.07, 0.27)	<0.0001	
Rad or Chemo only	45	39 (87%)	1.21 (0.56, 2.59)	0.6320	
Rad + Chemo	76	50 (66%)	0.57 (0.27, 1.21)	0.1422	
Other^1^	9	8 (89%)	–		
Gender					<0.0001
Female^1^	604	170 (28%)	–		
Male	670	269 (40%)	1.48 (1.22, 1.80)	<0.0001	
ECOG performance score					<0.0001
0, 1	1094	331 (30%)	0.27 (0.21, 0.33)	<0.0001	
2, 3, 4^1^	156	101 (65%)	–		
Smoking cessation					0.0001
Quit^1^	1217	411 (34%)	–		
Kept smoking	57	28 (49%)	2.10 (1.43, 3.09)	0.0002	
Pack-years smoked					<0.0001
0–20	426	117 (27%)	0.60 (0.48, 0.75)	<0.0001	
20–40	280	101 (36%)	0.87 (0.69, 1.11)	0.2641	
>40^1^	563	221 (39%)	–		
Any other cancer					0.6813
Yes^1^	174	78 (45%)	–		
No	983	318 (32%)	1.05 (0.82, 1.36)	0.6813	
Weight loss of 5% in past 6 months					0.0958
No^1^	1070	374 (35%)	–		
Yes	163	48 (29%)	0.78 (0.57, 1.05)	0.0969	
Tumor diameter (cm)	1274	439 (34%)	1.12 (1.08, 1.15)	<0.0001	<0.0001

QOL, quality of life; ECOG, Eastern Cooperative Oncology Group.

1 Reference group.

The MV analysis revealed that all these factors were significant predictors of survival. Patients reporting a QOL deficit had significantly worse survival rates even after adjusting for other known prognostic variables (*P* < 0.0001, HR = 1.84 with a 95% CI 1.44–2.35). See Table3 for MV Cox proportional hazard model results. The 5-year OS was reduced by greater than one half for patients reporting QOL deficits (29.9% vs. 62.8%); ECOG performance status of >1 (24.3% vs. 61.8%) and continued smoking (28.2% vs. 58.6%).

**Table 3:** Multivariate Cox regression model survival analysis using first QOL assessment

Effect	Hazard ratio	95% hazard ratio confidence limits		*P*-value
QOL (vs. > 50)^1^				
Deficit (QOL ≤ 50)	1.841	1.440	2.354	<0.0001
Age, years (vs. ≥ 80)^1^				
<60	0.395	0.278	0.562	<0.0001
60–69.999	0.489	0.351	0.680	<0.0001
70–79.999	0.795	0.582	1.085	0.1479
Sex (vs. male)^1^				
Female	0.782	0.639	0.957	0.0169
ECOG performance status (vs. 2, 3, 4)^1^				
0, 1	0.448	0.344	0.585	<0.0001
Smoking cessation (vs. kept smoking)^1^				
Quit	0.496	0.336	0.733	0.0004
Tumor size (>2 cm)^1^				
≤2 cm	0.702	0.563	0.874	0.0016
Regional nodal involvement (vs. metastasis in contralateral mediastinal)^1^				
No nodal metastases	0.259	0.156	0.428	<0.0001
In ipsilateral peribronchial and/or ipsilateral hilar nodes	0.402	0.231	0.701	0.0013
In ipsilateral mediastinal and/or subcarinal nodes	0.574	0.345	0.954	0.0323
Distant metastasis (vs. presence)^1^				
Absence	0.274	0.204	0.368	<0.0001

QOL, quality of life; ECOG, Eastern Cooperative Oncology Group.

1 Reference group.

**Figure 1:**
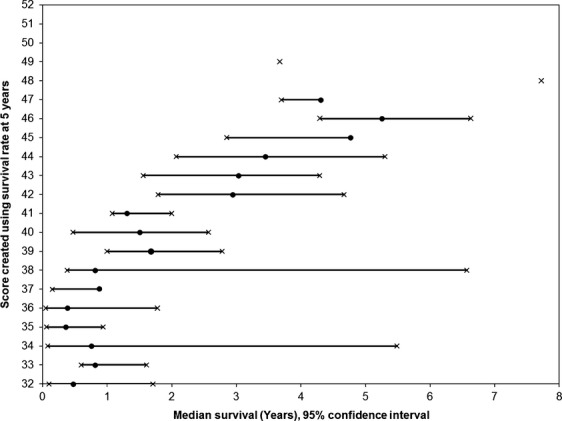
Median survival for patients with each total numeric score.

**Figure 2:**
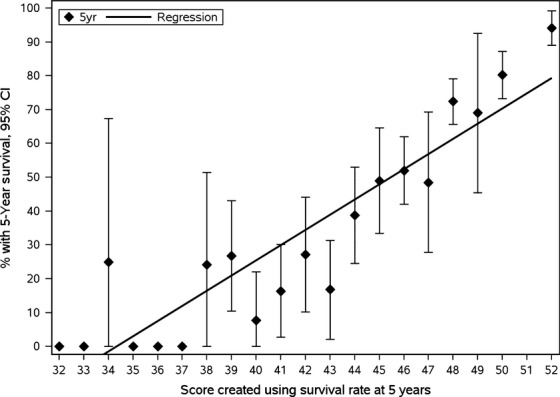
Total score and the corresponding 5-year survival.

The score was calculated for each prognostic factor by dividing the 5-year survival rate in percent by 10. Individual score ranged from 1 to 7 points. High 5-year survival rates correlated to higher scores (Table4). The total scores were calculated for each patient based on the sum of the scores for each prognostic factor and ranged from 32 to 52 points. Kaplan–Meir survival estimates by total score are shown in Table5. Figure1 shows the median survival for each corresponding total score. Figure2 shows the total score and the corresponding 5-year survival rates. The 5-year OS by different total scores are categorized in Table6. Within category 4, patients with a low total score of 32 to 37 had a significantly worse OS (*P* < 0.0001, HR = 29.06 with a 95% CI 18.49–45.66) compared to patients with a high total score (50–52). All categorization schemes demonstrated successful prognostic power (Table6). Category 4 divided patients into groups with total scores of 32–37, 38–43, 44–47, 48–49, and 50–52 with 5-year OS rates of 8%, 20%, 48%, 72%, and 85%, respectively (*P* < 0.0001).

**Table 4:** Five-year survival rates and the corresponding score

Variable	Five-year survival, %	Score
QOL		
Non-deficit (>50)	63	6
Deficit (QOL ≤ 50)	30	3
Age, years		
<60	67	7
60–69.999	65	7
70–79.999	48	5
≥80	38	4
Sex		
Female	65	7
Male	51	5
ECOG performance score		
0, 1	62	6
2, 3, 4	24	2
Smoking cessation		
Quit	59	6
Kept smoking	28	3
Tumor size		
≤2 cm	69	7
>2 cm	50	5
Regional nodal involvement		
No nodal metastases	65	7
In ipsilateral peribronchial and/or ipsilateral hilar nodes	46	5
In ipsilateral mediastinal and/or subcarinal nodes	33	3
Metastasis in contralateral mediastinal nodes	13	1
Distant metastasis		
Absence	61	6
Presence	11	1

QOL, quality of life; ECOG, Eastern Cooperative Oncology Group.

**Table 5:** Median survival time, 5-year survival, and the corresponding score using survival rate at 5 years to create the score

Total score	*N* (total = 1250)	Median survival, years (95% CI)	Five-year survival rate (%)
32	6	0.48 (0.10, 1.71)	0.0
33	4	0.82 (0.60, 1.61)	0.0
34	4	0.76 (0.08, 5.49)	25.0
35	7	0.36 (0.06, 0.94)	0.0
36	9	0.39 (0.05, 1.78)	0.0
37	14	0.89 (0.15, NA)	0.0
38	11	0.82 (0.38, 6.57)	24.2
39	41	1.68 (1.0, 2.78)	26.8
40	19	1.51 (0.47, 2.57)	7.7
41	59	1.31 (1.08, 2.0)	16.4
42	43	2.95 (1.79, 4.67)	27.2
43	61	3.04 (1.56, 4.29)	16.8
44	69	3.45 (2.07, 5.30)	38.8
45	81	4.77 (2.85, NA)	49.0
46	157	5.26 (4.30, 6.63)	52.0
47	52	4.31 (3.70, NA)	48.5
48	250	NA (7.73, NA)	72.4
49	24	NA (3.68, NA)	69.1
50	224	NA (NA, NA)	80.3
51	–	–	–
52	115	NA (NA, NA)	94.1

Variables used: Quality of Life, Age, Sex, Eastern Cooperative Oncology Group performance status (PS), Smoking Cessation, Tumor Size, Regional Nodal Involvement, Distant Metastasis.

**Table 6:** Overall survival by different total score categories

Variable	*N*	Events	Median years	Five-year survival % (95% CI)	log-rank *P*-value	Cox univariate hazard ratio (95% CI)	Cox univariate Wald *P*-value	Cox Univariate Score *P*-value
Total score category 1					<0.0001			<0.0001
32–36	30	29 (97%)	0.5	3.6% (0.0%, 10.5%)		33.46 (20.65, 54.21)	<0.0001	
37–38	25	17 (68%)	0.8	23.2% (4.3%, 42.2%)		17.30 (9.81, 30.50)	<0.0001	
39–41	119	81 (68%)	1.5	19.0% (9.8%, 28.1%)		12.46 (8.54, 18.16)	<0.0001	
42–44	173	92 (53%)	3.0	29.0% (20.1%, 37.9%)		6.75 (4.68, 9.74)	<0.0001	
45–47	290	105 (36%)	5.3	50.5% (42.7%, 58.3%)		3.87 (2.70, 5.54)	<0.0001	
48–49	274	66 (24%)	NA	72.1% (65.6%, 78.6%)		1.98 (1.34, 2.91)	0.0006	
50–52^1^	339	42 (12%)	NA	84.7% (79.6%, 89.8%)		–		
Total score category 2					<0.0001			<0.0001
32–35	21	20 (95%)	0.5	5.3% (0.0%, 15.4%)		68.65 (28.89, 163.11)	<0.0001	
36–38	34	26 (76%)	0.8	15.2% (1.7%, 28.7%)		42.31 (18.31, 97.79)	<0.0001	
39–41	119	81 (68%)	1.5	19.0% (9.8%, 28.1%)		25.45 (11.74, 55.19)	<0.0001	
42–44	173	92 (53%)	3.0	29.0% (20.1%, 37.9%)		13.81 (6.40, 29.79)	<0.0001	
45–47	290	105 (36%)	5.3	50.5% (42.7%, 58.3%)		7.92 (3.68, 17.02)	<0.0001	
48–50	498	101 (20%)	NA	75.7% (71.0%, 80.5%)		3.39 (1.58, 7.29)	0.0018	
51–52^1^	115	7 (6%)	NA	94.1% (89.0%, 99.2%)		–		
Total score category 3					<0.0001			<0.0001
32–35	21	20 (95%)	0.5	5.3% (0.0%, 15.4%)		67.82 (28.55, 161.15)	<0.0001	
36–39	75	53 (71%)	1.1	21.0% (9.7%, 32.3%)		28.78 (13.06, 63.42)	<0.0001	
40–43	182	112 (62%)	2.0	18.6% (10.7%, 26.4%)		19.38 (9.02, 41.65)	<0.0001	
44–47	359	139 (39%)	4.4	48.3% (41.5%, 55.2%)		8.61 (4.03, 18.39)	<0.0001	
48–50	498	101 (20%)	NA	75.7% (71.0%, 80.5%)		3.39 (1.58, 7.29)	0.0018	
51–52^1^	115	7 (6%)	NA	94.1% (89.0%, 99.2%)		–		
Total score category 4					<0.0001			<0.0001
32–37	44	37 (84%)	0.6	8.3% (0.0%, 17.1%)		29.06 (18.49, 45.66)	<0.0001	
38–43	234	148 (63%)	1.9	20.0% (13.0%, 27.0%)		9.97 (7.05, 14.09)	<0.0001	
44–47	359	139 (39%)	4.4	48.3% (41.5%, 55.2%)		4.21 (2.98, 5.95)	<0.0001	
48–49	274	66 (24%)	NA	72.1% (65.6%, 78.6%)		1.98 (1.34, 2.91)	0.0006	
50–52^1^	339	42 (12%)	NA	84.7% (79.6%, 89.8%)		–		

1 Reference group.

Sensitivity analyses using bootstrap approach provided results that were similar to the original analyses. In the MV model validation, the percent of time the variables were included in the bootstrapped model were 100% for overall QOL, 100% for age, 100% for ECOG performance status, 100% for regional nodal involvement, 100% for distant metastasis, 97% for smoking cessation, 95% for tumor size, and 78% for sex. In score validation, the median and mean survival rates at 5 years from bootstrapped samples only differ by 0.1% to 3.2% from the 5-year survival rates on original samples (Table7).

**Table 7:** Summary comparison of survival rates at 5 years for 1000-iterations of bootstrap on 1274 subjects and original samples

Variable	Summary statistics for 1000-iterations of bootstrap on 1274 subjects					Survival rates at 5 years on original samples (%)
	Median (%)	Minimum (%)	Maximum (%)	Mean (%)	Standard deviation (%)	
QOL						
Non deficit (QOL>50)	62.9	56.6	68.8	62.8	1.8	62.8
Deficit (QOL ≤ 50)	29.8	14.8	43.5	29.8	4.6	29.9
Age, years						
<60	67.1	55.8	77.1	67.1	3.1	67.1
60–69.999	64.9	54.2	73.5	64.9	2.9	64.6
70–79.999	48.2	36.6	56.8	48.2	3.1	48.2
≥80	38.1	18.3	59.4	38.2	6.0	38.2
Gender						
Female	64.9	55.7	73.7	64.8	2.5	64.8
Male	51.3	43.8	59.6	51.4	2.4	51.3
ECOG performance score						
0, 1	61.8	55.1	68.6	61.8	1.8	61.8
2, 3, 4	24.3	13.4	41.0	24.5	4.3	24.3
Smoking cessation						
Quit	58.7	53.3	63.5	58.6	1.8	58.6
Kept smoking	29.1	6.9	66.6	29.5	10.0	28.2
Tumor size (cm)						
≤2 cm	69.2	61.2	78.1	69.2	2.7	69.2
>2 cm	50.4	43.8	56.7	50.4	2.2	50.4
Regional nodal involvement						
No nodal metastases	65.4	58.8	71.7	65.3	2.0	65.3
In ipsilateral peribronchial and/or ipsilateral hilar	45.9	23.4	66.5	45.8	5.7	46.0
In ipsilateral mediastinal and/or subcarinal	33.3	20.5	45.1	33.4	4.0	33.2
Metastasis in contralateral mediastinal	14.7	3.6	47.6	15.7	7.5	12.5
Distant metastasis						
Absence	60.9	55.0	66.0	60.9	1.8	60.9
Presence	10.7	1.7	27.5	11.0	4.3	10.9

QOL, quality of life; ECOG, Eastern Cooperative Oncology Group.

## Discussion

Lung cancer is a significant health care problem as the leading cause of cancer deaths 1. A clear understanding of the various prognostic factors is important for a number of reasons. Physicians can use this information to give patients and their families' realistic impressions of survival. Also, the ability to predict survival can help tailor therapy to individual patients.

Proper trial design requires a clear understanding of critical prognostic factors. This is important as imbalances in the distribution of pretreatment prognostic factors can influence survival as much as treatment. Thus, imbalances in the distribution of various prognostic factors between treatment groups can bias the outcome and lead to incorrect conclusions. This can create situations where effective therapies appear useless and ineffective therapies appear useful. Thus, one important use of this scoring system is in the proper stratification of patients in future trials. This study was undertaken to use many significant prognostic factors to create a scoring system that can better predict survival than was previously possible for NSCLC patients.

This score can also be used to define eligibility criteria in trials designed for specific patient populations. For example, the criteria for defining high-risk populations in lung cancer generally only rely on stage, weight loss, and performance status 8. This analysis allows investigators to use more prognostic factors and understand the influence of them individually and collaboratively on patient survival.

Many investigators have evaluated prognostic factors in patients with nonmetastatic (M0) NSCLC. Jeremic et al. 5 identified female sex, performance status, weight loss, stage, histology, inter-fraction interval, and treatment as prognostic factors in stage III NSCLC. Mosvas identified QOL as the sole independent prognostic factor in stage III NSCLC patients 9. Additionally, other investigators have identified stage, radiotherapy technique, hoarseness, malaise, erythropoietin, and estrogen receptors in tumor cells as prognostic factors in patients without distant metastases 10–12. The present study identified age, diameter of the primary tumor, regional nodal involvement, distant metastases, overall QOL, treatment, ECOG performance score and smoking cessation as independent prognostic factors for survival.

Wigren developed a prognostic index based on a patient cohort with inoperable stages I–IIIb NSCLC. The five factors identified were disease extent, clinical symptom score by Feinstein, performance status, tumor size, and hemoglobin level. These key prognostic variables of the index had equal impact on survival. Thus, based only on the number of adverse factors, each patient falls into one of the six possible prognostic groups. All five factors were significantly predictive of survival and the inclusion of the other known prognostic variables in the MV analyses did not result in any further improvement. Patients with three or more risk factors had a 2-year survival rate of less than 2%, whereas the 17 patients (8%) with no risk factors had a survival of 53%. Wigren concluded that this information could be used to guide management strategy, help to design new treatment strategies, and facilitate the comparison of different studies 13,14. However, this prognostic index was based only on patients with inoperable stages I-IIIb NSCLC and is not applicable to the other patients groups as is the scoring system developed in the present analysis.

Hoang, Finklestein, Paesmans, and Albain examined patients with stage IV disease and found the following factors to be of prognostic importance: performance status, sex, weight loss, metastases to specific locations (skin, bone, liver), number of metastatic sites, advanced age, and certain laboratory findings (abnormal calcium, white blood counts, lactate dehydrogenase, and anemia) 15–18. Mandrekar et al. 19 went further to develop a mathematical model to predict the survival of patients with stage IV NSCLC. This formula was based on various prognostic factors including performance status, basal metabolic index (BMI), hemoglobin levels, and white blood count.

In a previous Mayo study, Sloan et al. 4 found survival was associated with QOL, performance status, age, smoking history, sex, treatment factors, and stage of disease in a large cohort of patients with all stages of disease. The emphasis of the Sloan et al. study was to define the importance of QOL as independent prognostic factor in NSCLC. The prognostic factors identified in both of these Mayo studies were consistent with those previously reported in the literature. Additionally, the cohort identified by Sloan et al. was further updated and analyzed in this study to develop this Mayo Score for NSCLC which could be used to predict 5-year survival based on a NSCLC patient's individual characteristics.

While the prognostic factors identified in the current study have been previously reported, a scoring system for patients with all stages of NSCLC has not been reported or widely adopted. One weakness of this analysis is the retrospective methodology that may have introduced unforeseen biases. However, the bootstrapping analyses revealed high consistency, lending credence to the content validity of the scoring system. This study included a primarily white population who were robust enough to seek care at a large tertiary care facility introducing potential bias. Another limitation of this study is that only 81 (6%) of the 1, 274 patients had metastatic disease which is lower than the general population of US lung cancer patients 2. The results for small subpopulations must be interpreted with care. For example, the confidence interval estimators for tiny populations are statistically quite large.

This study was undertaken to use independent pretreatment prognostic factors to create a single scoring system that can predict survival for all NSCLC patients. The score is based on data that is easily obtained during the evaluation of lung cancer patients. The only factor within this system that is not collected routinely during the evaluation of NSCLC patients is the QOL score that can be collected in a minute or so by having each patient judge the overall quality of their lives with a single 0–100 scale. This Mayo Score can provide accurate estimations of patient survival, aid in proper stratification in future trial design, help tailor therapy to individual patients, and identify patients for high-risk trials. Optimally, this scoring system should be further validated with other data sets to confirm its utility. Additionally, we expect this score will be refined over time as the molecular nature of NSCLC is more fully elucidated, better therapies are developed, and patient survival improves.

## Conflict of Interest

None declared.
